# Evaluation of the Sexual Health Behaviors of Black Male Adolescents and Young Adults Through Social Media Platforms: Web-Based Survey Study

**DOI:** 10.2196/19219

**Published:** 2020-09-22

**Authors:** Jade Burns, Keith Johnstone, Tanaka Chavanduka, Cornelius Jamison, Valery Pena, Rob Stephenson, Lynae Darbes

**Affiliations:** 1 Department of Health Behavior and Biological Sciences University of Michigan School of Nursing Ann Arbor, MI United States; 2 Ross School of Business University of Michigan Ann Arbor, MI United States; 3 Center for Sexuality and Health Disparities University of Michigan School of Nursing Ann Arbor, MI United States; 4 Department of Family Medicine Institute for Healthcare Policy & Innovation University of Michigan Ann Arbor, MI United States; 5 The College of Arts and Sciences Cornell University Ithaca, NY United States; 6 Department of Systems, Population and Leadership Center for Sexuality and Health Disparities University of Michigan School of Nursing Ann Arbor, MI United States

**Keywords:** social media, survey, adolescent, young adult, Black, males, sexual health, service delivery

## Abstract

**Background:**

Social media platforms such as Facebook, Instagram, and Twitter, which have millions of users who interact and communicate every day, have been effective in promoting sexual health interventions and in disseminating reproductive health education. They have also been shown to be useful in health promotion and have been used to track several key metrics (eg, comments, posts) among users of all demographics. However, there is a lack of research on the impact and reach of these social media platforms as a community-based tool for disseminating sexual health information and for increasing engagement among Black adolescents and young adults, which is a targeted high-risk population.

**Objective:**

The purpose of this study was to determine the social media platforms and banner advertisements that affected engagement among Black male adolescents and young adults in participating in web-based health surveys.

**Methods:**

A web-based survey was conducted from March 2019 to July 2019 to assess sexual health and health behaviors in a convenience sample of Black male adolescents and young adults in the age range of 18-24 years (N=170). Social media metrics from Facebook, Instagram, and Twitter were monitored. This cross-sectional survey comprised several categories, including basic personal information, drug-related risk behaviors, health care, sexual reproductive health questions, attitudes, norms, and perceived control, mental health, violence-related risk behaviors, and social media preferences.

**Results:**

Social media advertisements on the Black Male Opinion survey reached approximately 146,412 individuals. Our primary finding of the web-based survey engagement was that referral (eg, group chat, indirect social media sharing) led to as the greatest proportion of recruitment, with Twitter and YouTube as the preferred sites to receive sexual health information.

**Conclusions:**

Recognizing the variety of technologies being used among Black male young adults and adolescents can help the community, researchers, and health care providers understand the web-based engagement of this high-risk population. This information may also promote culturally sensitive, customized marketing on sexual health information for this population.

## Introduction

Internet and social media use are universal among adolescents and young adults in the United States, with 80% having access to either a smartphone or broadband internet [1]. Moreover, about two-thirds of the Americans claim that internet access is essential for accessing personal health information [2,3]. An increasing number of health care systems are adapting to this new “social media age” and integrating these platforms into their services. Various hospitals use social media to increase engagement, marketing, and their impact on local communities [3,4]. In addition, social media platforms have increasingly become a source of data to the general public and a means of spreading health awareness, promoting screenings, and reducing health disparities [5]. Major health care systems such as the Mayo Clinic have begun beta testing the impact of social media by creating Twitter accounts and by using analytics to track engagement and the impact on their marketing [6]. This shift toward a more social media–friendly approach in the health care system in general, particularly in primary care, is a result of the need to adapt to new technologies and to reach those disproportionately affected by health problems due to socioeconomic factors. 

Young Black males, specifically the emerging adulthood population, in the age range of 18-24 years, have a substantially greater need for sexual reproductive health services and are at a higher risk of contracting sexually transmitted infections (STIs) than other adolescents and young adult populations [7-13]. Likewise, the sexual reproductive health needs of this population are often underaddressed and insufficiently understood in clinical settings [7]. One possible cause for these disparities is the lack of access to sexual health resources such as education and condoms. To increase access in hard-to-reach populations, social media platforms have been used to promote health resources and have been slowly incorporated into health care. Addressing disparities in sexual health among young Black males could become more feasible with the use of these platforms [14-16].

Social media platforms such as Facebook, Instagram, and Twitter, which have millions of users who interact and communicate every day, have been effective in promoting sexual health interventions and in the spread of reproductive health education [17,18]; some examples of promoting these interventions are by improving STI knowledge, encouraging people to undergo testing or screening for HIV/STIs, and using social media as a resource for STI education [19]. These platforms have also been shown to be useful in health promotion and have been used to track several key social media metrics (eg, comments, posts) among users of all demographics. However, there is a lack of research on the impact and reach of these social media platforms as a community-based tool for sexual health promotion and on the increasing engagement of social media use among Black adolescents and young adults, which is a targeted high-risk population [20,21]. We recently surveyed young heterosexual Black males across the United States. We sought to learn the extent to which young Black males engage in taking web-based surveys via social media. Thus, the purpose of this study was to determine the social media platforms and banner advertisements that affected the engagement of Black male adolescents and young adults in participating in web-based health surveys. Social media metrics such as “likes,” “impressions” (number of times an advertisement is shown on a social media page that may not be unique to individuals), and “link clicks” were used to assess the impact of the engagement. Recognizing the variety of technologies being used by this population can help the community, researchers, and health care providers understand how Black young adults and adolescents can be engaged through web-based services. This information can promote culturally sensitive customized marketing on sexual health information for this population.

## Methods

### Recruitment

Project Black Male Opinion was a web-based survey disseminated via the study staff through mass email listservs, Facebook, Instagram, and Twitter. Dissemination of the survey was also supported by collaborative partnerships that included a Federally Qualified Health Center Organization in Detroit and the University of Michigan Center for Sexuality and Health Disparities. The data reported here is the first subanalysis of a larger survey to collect information to understand the association between social media use and sexual health care among Black male adolescents and young adults. The goal of the larger study was to (1) determine the optimal social media sites to disseminate sexual health information to this population and (2) build a registry of zip codes to examine cross-sectional data on sexual reproductive health and risk behaviors (eg, substance abuse, violence) in young Black males beyond the local Detroit community. A convenience sample was used to recruit Black male adolescents and young adults who met certain practical criteria (eg, geographic criteria, web-based accessibility, willingness to participate) [22] to participate in the web-based survey. The study advertisements were designed to recruit young Black males between the ages of 18 years and 24 years who expressed a “multicultural affinity” through their profile self-identification or posted content pertaining to being Black and male.

This study used Facebook, Instagram, and Twitter to promote social media advertisement campaigns. A previous study indicated that these sites are best used to promote condom use education among young Black males [23]. Another recent study showed that these sites are frequently used by Black youth and are cost-effective [24]. Facebook analytics were used to track and analyze the audience and their engagement with each post. Since Facebook owns Instagram, the study team was able to review all the posts from both social media platforms simultaneously. On the Facebook page, an insight tab allowed the study team to analyze the “actions” on the page. The actions included the frequency of reviewing or previewing a post, the number of individuals that “liked” the page, user engagement (a combination of likes, comments, and shares), and the number of times the individuals saw the post across their social media timelines (reach) [25,26]. Each week, social media analytics were reviewed for the total reach on each social media platform, including the number of “impressions” and “likes” and the number of “clicks” on the survey link. The goal of this descriptive study was to collect a maximum of 300 survey responses. This web-based survey was open from March 2019 to July 2019, gathering a total sample response of 170 respondents who answered the survey on their sexual health care and health behavior. Recruitment materials consisted of designed advertisements that used stock images of young Black males on Facebook, Instagram, and Twitter websites (including our community and academic partner websites) and directed participants to the Qualtrics survey tool. The advertisements indicated the purpose of the study and the inclusion criteria for participation. The survey website to which the participants were directed contained additional information about the details of the survey, the contact information of the study staff, and informed consent.

### Study Population

Our study included participants who were (1) 18-24 years of age (2) self-identified as Black or African American (3) males, and (4) living in the United States. We excluded participants who did not reside within the United States, who were under the age of 18 years, and did not self-identify as African American or Black.

### Survey Development and Analysis

Social media metrics from Facebook, Instagram, and Twitter were monitored. This cross-sectional survey comprised several categories, including basic personal information, drug-related risk behaviors, health care plans (eg, current insurance plan), sexual reproductive health questions (eg, last sexual encounter, number of partners, testing, clinic utilization), attitudes, norms, and perceived control (eg, condom use behaviors, HIV/STI transmission), mental health (eg, opinion of self, safe space), violence-related risk behaviors (eg, fighting, physically threatened), and social media preferences. Questions were selected from the Youth Risk Behavior Surveillance System Survey, the Centers for Disease Control and Prevention, and the National Health and Nutrition Examination Survey [27,28]. The survey was administered using the Qualtrics survey programming software. Before administration, closed beta testing was used to review the content and the clarity of the questions. Participants accessed the survey by clicking on a link in the banners and other photo advertisements targeted at young Black males through a personal social media marketing plan. Web-based electronic consent was obtained at the beginning of the survey. The institutional review board at the sponsoring institution approved all the survey and the study procedures.

For this analysis, we have described the process of recruitment, rates of enrollment, demographics of the eligible participants, and the associated costs across the social media platforms. Our outcome of interest was survey participation through branded social media advertisements. For the purpose of this analysis, participants were categorized as eligible once they provided consent to screen for eligibility, met eligibility criteria, and completed the survey. Additionally, we report the social media metrics (ie, link clicks, reach, and impressions) that generated the participant recruitment of our eligible sample and their demographic characteristics. All additional descriptive analyses were performed using the statistical software, that is, software for statistics and data science 15.0 (StataCorp).

## Results

In total, the Black Male Opinion social media advertisements reached approximately 146,412 individuals, generating 187,320 impressions, and resulting in 0.80% (1483/187,320) clicks. Of those individuals who clicked the advertisements, web-based electronic consent to screen for eligibility was obtained from 14.7% (218/1483) of the sample population. Of these individuals, 78.0% (170/218) started the survey, and 47.1% (80/170) of these individuals met the eligibility criteria. The reasons for ineligibility included not identifying as Black or African American (22/90, 24%), age greater than 24 years (47/90, 52%), and not male gender (12/90, 13%) (Table 1). The total cost for all paid advertising was US $1067.90 through a 5-month recruitment period. The cost can be broken down as follows: US $1.39 per click, US $4.90 per consent, and US $13.35 per eligible participant.

**Table 1:** Descriptive statistics of the participants and the demographic variables (N=170).

Characteristics			Ineligible (n=90)^a^, n (%)		Eligible (n=80), n (%)
**Age (years)**					
	Under 18	12 (13)		N/A^b^	
	18-20	16 (18)		39 (49)	
	21-22	12 (13)		22 (27)	
	23-24	3 (3)		19 (24)	
	25+	47 (52)		N/A	
**Hispanic or Latino**					
	Yes	12 (28)		10 (12)	
	No	31 (72)		70 (87)	
**Identify as Black or African American**					
	Yes	21 (49)		80 (100)	
	No	22 (51)		N/A	
**Gender**					
	Male	3 (20)		80 (100)	
	Female	5 (33)		N/A	
	Transgender	7 (47)		N/A	
**Sexual orientation**					
	Straight	10 (59)		60 (75)	
	Gay	1 (6)		13 (16)	
	Bisexual	3 (18)		5 (6)	
	Preferred not to say	1 (6)		2 (2)	
	Other	2 (12)		N/A	
**Has health insurance**					
	Yes	6 (75)		67 (84)	
	No	2 (25)		13 (16)	
**Education**					
	Up to high school	4 (27)		13 (16)	
	Some college/technical degree	9 (60)		43 (54)	
	College/Graduate school	2 (13)		24 (30)	

^a^It was not mandatory for the ineligible participants to answer all the questions related to demographics. Therefore, the percentages in this column were calculated on the basis of those who responded.

^b^Not applicable.

The participant age distribution comprised mostly of males aged 18-20 years (39/80, 49%), 21-22 years (22/80, 27%), and 23-24 years (19/80, 24%) years (Table 2). The mean age of the final sample was 21 years old, with a majority of the participants being 19 years old. A smaller proportion of the participants was identified as being Hispanic or Latino (10/80, 12%). The majority of the sample was identified as straight in sexual orientation (60/80, 75%), had health insurance (67/80, 84%), and had some college or technical education (43/80, 54%).

**Table 2:** Demographics of the eligible participants in each recruitment platform (n=80).

Demographic characteristics		Total, n=80, n (%)	Referral, n=27, n (%)	Facebook, n=14, n (%)	Instagram, n=8, n (%)	Missing, n=18, n (%)	Email, n=9, n (%)	Other^a^ (n=2), n (%)
**Age (years)**								
	18-20	39 (49)	13 (48)	4 (29)	7 (87)	8 (44)	5 (56)	2 (100)
	21-22	22 (27)	9 (33)	5 (36)	1 (12)	4 (22)	2 (22)	N/A^b^
	23-24	19 (24)	5 (18)	5 (36)	0 (0)	6 (33)	2 (22)	N/A
**Hispanic or Latino**								
	Yes	10 (12)	7 (26)	1 (7)	1 (12)	1 (6)	N/A	N/A
	No	70 (87)	20 (74)	13 (93)	7 (87)	17 (94)	9 (100)	2 (100)
**Sexual orientation**								
	Straight	60 (75)	19 (70)	9 (64)	6 (75)	15 (83)	8 (89)	2 (100)
	Gay	13 (16)	4 (15)	3 (21)	2 (25)	3 (17)	1 (11)	N/A
	Bisexual	5 (6)	3 (11)	2 (14)	N/A	N/A	N/A	N/A
	Preferred not to say	2 (2)	1 (4)	N/A	N/A	N/A	N/A	N/A
**Has health insurance**								
	Yes	67 (84)	24 (89)	11 (79)	7 (87)	14 (78)	8 (100)	2 (100)
	No	13 (16)	3 (11)	3 (21)	1 (12)	4 (22)	N/A	N/A
**Education**								
	Up to high school	13 (16)	2 (7)	4 (29)	1 (12)	4 (22)	1 (11)	1 (50)
	Some college/technical degree	43 (54)	15 (56)	7 (50)	5 (62)	10 (56)	5 (56)	N/A
	College/Graduate school	24 (30)	10 (37)	3 (21)	2 (25)	4 (22)	3 (33)	1 (50)

^a^Includes 2 more from an unspecified web-based advertisement.

^b^Not applicable.

Across platforms, our primary finding for web-based survey engagement was that referral (eg, group chat, indirect social media sharing) provided the greatest proportion of recruitment (27/80, 34%), while other methods such as web-based spaces that we were not able to capture (2/80, 0%) and Instagram (8/80, 10%) obtained the lowest rate of recruitment. The method of recruitment was missing for individuals who did not provide a response (18/80, 24%). Those with missing recruitment platforms may fall into the other categories, potentially undercounting the frequencies of the other platforms. Users reported Twitter (24/80, 39%) and YouTube (27/80, 34%) as being the best platforms to receive sexual health information. Overall, different sites produced different results in engagement and cost. Table 3 indicates the sample banner advertisement engagement across social media platforms and the associated costs per participant.

**Table 3:** Subset of recruitment advertisement performance by platform.

Advertisement name	Facebook				Instagram			
	Reach (n)	Impression (n)	Engagement^a^ (n)	Cost per engagement (US $)	Reach (n)	Impression (n)	Engagement (n)	Cost per engagement (US $)
Your Opinion Counts	42,958	47,207	252	0.74	N/A	N/A	N/A	N/A
Your Opinion Matters	15,880	21,690	98	0.75	N/A	N/A	N/A	N/A
Be more informed	5788	5865	59	0.64	N/A	N/A	N/A	N/A
Be more visible	1181	1213	377	0.09	15,180	18,768	96	0.87
Help us change	N/A	N/A	N/A	N/A	4402	5388	78	0.45
Be more together	3245	3717	30	0.78	3300	3772	17	1.22
Be more in sync	630	639	59	0.28	416	422	19	0.50

^a^Engagement is defined as any user interaction with an advertisement, and it may include a link click, comment, or like.

^b^Not applicable.

## Discussion

### Principal Findings

We conducted a web-based survey to determine the social media platforms and the banner advertisements that affected the engagement of self-identified Black adolescents and young adults in participating in web-based surveys. In this web-based survey, 80 eligible individuals completed the Black Male Opinion social media survey. Overall, we found that young Black males who participated in this survey joined via referral through other social media outlets (eg, friends sending links via Facebook Messenger or Instagram Direct Message). Empirically, this population engaged through Facebook most frequently via paid web banner advertisements. However, in the survey responses, participants reported a preference for Twitter and YouTube as venues for receiving sexual health information. The paid banner advertisements resulted in reaching approximately 150,000 individuals within the defined demographic over 5 months.

Previous studies have shown that social media platforms have the potential to promote safe sex practices and STI prevention among adolescents and young adults engaging in high-risk behaviors [20,29]. The response from this engagement in this study shows the possibility of reaching a large untapped population. With these findings, there is also the potential for targeted use of one of the identified social networks that this target audience prefers as well as for a comparison of the distribution and uptake of this survey in other social media campaigns with adolescents and young adults and health behaviors. By using this marketing strategy, more culturally sensitive and customized health information may be created to promote health awareness such as STI-screening locations, accurate sexual health education, and condom availability for this demographic. Moreover, using this type of web-based engagement may be useful in removing barriers such as transportation and face-to-face engagement (eg, distrust in the medical system, stigma, lack of community-based resources, lack of knowledge) [15,30,31]. The social media site that would be best suited as a community-based tool for the generalized population is still unclear in this study. However, from our sample, there is an indication that YouTube and Twitter are viable methods for promoting sexual health information and education to young Black males. Future research would need to affirm these findings by designing a pilot study that directly compares these social media platforms to weigh the benefits that each site would have to offer. This work is one step closer to potentially utilizing a more creative and culturally sensitive approach that may be included in the current health care system, more specifically in primary care. However, the ultimate goal is not only to engage young Black males but also to aid them in finding a safe space for navigating services and participating in conversations that are relevant about their identities and sexual health both in and outside of the clinic.

### Limitations

Our study has the following limitations. First, our sample size was small and therefore is not representative of the entire population. Offering an incentive may have enhanced the response rate [32]. Using a multimodal approach such as traditional recruitment methods (eg, word of mouth, flyers) in combination with other social media platforms (eg, Reddit, Snapchat) may have helped to cover broader demographics and reach a larger and more diverse pool of potential respondents [33]. Second, requests from social media sites to update banner advertisements due to incorrect wordings and noncompliance with advertisement policies consumed additional time for running and producing the advertisements. Reviewing advertisement policies before posting, such as Facebook’s brand or content that asserts or implies personal attributes such as gender identity or age, can help to create an appropriate user-friendly advertisement [34]. Setting up a personalized marketing plan with a marketing expert for the intended social media site can also help to specify design formats and optimize recruitment campaigns for future studies. Third, owing to budget constraints, it was difficult to use more than 3 types of social media platforms to market the advertisements. Finally, these data have limited generalizability to a broader population. Nonetheless, our findings from this study contribute to the currently limited information available about this population.

### Implications

There is an urgent need for health care researchers to focus on improving the health of adolescents and young adults to better engage vulnerable populations, particularly Black males who have a need for sexual reproductive health services. Since adolescents and young adults universally engage over social media platforms, health care researchers have the opportunity to creatively develop web-based sexual reproductive health services that attract adolescents and young adults. This engagement is defined in different ways such as observation (ie, viewing the advertisement), liking the advertisements, or clicking the links. Reaching communities via methods of web-based social media platforms is often dictated by the advertising policies that each social media site uses such as the advertisement review process, brand assets, steps to take if the advertisement is disapproved, and prohibited content [34]. As social media platforms become more complex in their use and development, so will their policies; thus, health care researchers, the community, and those interested in reaching adolescents and young adults must be flexible and creative in how they attract users and build engagement on these important topics. Black male adolescents and young adults will benefit from the adoption of these evolving platforms into the research methodologies. Providing ongoing evidence of the extensive social media platform use among adolescents and young adults will inform policies and effective primary care and hospital system changes that may improve sexual health access.

Black male adolescents and young adults have an increased rate of STIs and would benefit from adolescent-friendly clinical practices regarding their sexual health [35]. In addition to providing sexual reproductive health care (eg, sexual education, STI screening) to adolescents and young adults in the clinical setting, health care providers have the opportunity to lead this type of innovation of using social media platforms for creating a space to discuss sexual health and to use these platforms to engage in creatively marketing sexual reproductive health resources. Thus, health care providers can become thought leaders in clinical settings to promote change or to ensure that this innovative method is implemented in the face of resistance or among those who do not use these platforms. With this in mind, these platforms can potentially increase engagement in this population by providing web-based information about STI screening in their local communities, obtaining condoms, and about alternative response venues to direct individuals to come into the clinic and to schedule appointments with a health care professional [23].

### Conclusion

A critical strategy in the efforts to reduce sexual health disparities is understanding the social media platforms that Black male adolescents and young adults engage in. This includes understanding the difference between the platforms that individuals may access on a regular basis versus the platforms that individuals may access to seek sexual health information for their personal use. This study demonstrates that this population engages through Facebook most frequently via paid web-based banner advertisements and through direct messages on Facebook and Instagram, which is a private form of communication via social media. Additionally, creating sexual reproductive health information on preferred platforms (eg, Twitter, YouTube) could benefit this vulnerable population. As social media platforms evolve and become more integrated into our culture, health care providers and researchers need to find novel ways to improve their marketing efforts and engagement with Black male adolescents and young adults. Creating policies and methodologies aimed at incorporating and utilizing these social media platforms will be vital in increasing sexual reproductive health education, resources, and access for adolescents and young adults, thereby promoting future health equity initiatives in virtual spaces and improved health outcomes for this underserved population.

## Abbreviations

STI: sexually transmitted infection
